# Whole genome sequence of *Vibrio cholerae* NB-183 isolated from freshwater in Ontario, Canada harbors a unique gene repertoire

**DOI:** 10.1186/s12863-024-01204-2

**Published:** 2024-02-15

**Authors:** Opeyemi U. Lawal, Noah Bryan, Mitra Soni, Yanhong Chen, Melinda Precious, Valeria R. Parreira, Lawrence Goodridge

**Affiliations:** 1 Canadian Research Institute for Food Safety (CRIFS), University of Guelph, Guelph, ON N1G 2W1 Canada; 2 Bayview Secondary School, 10077 Bayview Ave, Richmond Hill, ON L4C 2L4 Canada

**Keywords:** Vibrio cholerae, Antimicrobial resistance, Virulence genes, Genomics, Comparative genomics, Water quality, Aquatic systems

## Abstract

**Objective:**

*Vibrio cholerae* is an enteric pathogen that poses a significant threat to global health. It causes severe dehydrating diarrheal disease cholera in humans. *V. cholerae* could be acquired either from consuming contaminated seafood or direct contact with polluted waters. As part of a larger program that assesses the microbial community profile in aquatic systems, *V. cholerae* strain NB-183 was isolated and characterized using a combination of culture- and whole-genome sequencing-based approaches.

**Data description:**

Here we report the assembled and annotated whole-genome sequence of a *V. cholerae* strain NB-183 isolated from a recreational freshwater lake in Ontario, Canada. The genome was sequenced using short-read Illumina systems. The whole-genome sequencing yielded 4,112,549 bp genome size with 99 contigs with an average genome coverage of 96× and 47.42% G + C content. The whole genome-based comparison, phylogenomic and gene repertoire indicates that this strain harbors multiple virulence genes and biosynthetic gene clusters. This genome sequence and its associated datasets provided in this study will be an indispensable resource to enhance the understanding of the functional, ecological, and evolutionary dynamics of *V. cholerae*.

## Objective

*V. cholerae* is a causative agent of human diarrheal disease cholera and poses a significant threat to global health [1, 2]. While *V. cholerae* is naturally found in aquatic systems [1, 3–5], its persistence in this environment is attributed to specific stress response and adaptation mechanisms that include biofilm formation on an array of surfaces, survival in different environmental conditions, as well as interaction with other organisms in such environment [2]. *V. cholerae* is also a foodborne pathogen that could be acquired either from consuming undercooked or raw seafoods or a direct contact with polluted waters [4, 5]. As part of a larger study that assesses the microbial community profile and tracks antimicrobial resistant and pathogenic clones of bacteria in aquatic systems [6–9], we isolated *V. cholerae* strain NB-183 from a recreational freshwater lake. The objective of this study is to report the characterized *V. cholerae* NB-183 strain using whole genome sequencing-based approach. We also provided an extensive genetic background and gene content analysis of this strain.

Water sample was collected in the Fall of 2023 from a recreational freshwater Kettle Lake in Ontario, Canada (43.9486°N, 79.4352°W). To concentrate and detect bacteria in the water sample, 3 mL of nanobeads (Ceres Nanosciences) was added to 5 L of lake water sample and stirred at room temperature for 30 min. Thereafter, beads were collected using a 5-micron sock filter. An aliquot (100 uL) was spread onto MacConkey agar plates and incubated overnight at 37 °C. Pure colonies of differing morphologies were transferred onto a fresh tryptic soy agar (TSA) plate. Phenotypic identification was performed using VITEK® (bioMérieux Canada).

## Data description

DNA extraction and whole-genome sequencing of a pure colony was performed as previously described [6, 7]. Briefly, genomic DNA was extracted using the DNeasy blood and tissue kit (Qiagen, Hilden, Germany) according to the manufacturer’s instructions. DNA libraries were prepared using the DNA prep tagmentation kit and IDT for DNA/RNA unique dual (UD) indexes from Illumina. 2 × 150-bp paired-end sequencing was performed on the Illumina MiniSeq system. Raw reads were preprocessed with FastQC v0.11.9 (https://github.com/s-andrews/FastQC) and trimmed using Trimmomatic v0.39 [10]. Reads with Phred scores of ≥ 30 was assembled *de novo* using SKESA v2.4.0 [11]. The assembly quality and genome completeness was assessed using QUAST v5.2 [12] and BUSCO v5.5 [13]. Sequence type (ST) assignment was performed using the multilocus sequence type (MLST) database [14]. Genome annotation was performed using the NCBI Prokaryotic Genome Annotation Pipeline v6.6 [15]. Resistome and virulome were identified using CARD [16] and VFDB [17, 18], respectively using minimum coverage of 70% and minimum identity of 90%. Plasmids were identified using MOB-suite v3.1 [19] while PHASTER [20] was used to detect phage regions in the draft genome. Biosynthetic gene clusters were assessed using web-based antiSMASH v7 [21]. Default parameters were used for all bioinformatics pipelines except where otherwise stated.

A total of 1,477,996 paired-end reads were obtained from sequencing isolate NB-183 (Dataset 1, [22]). While the VITEK result was inconclusive, isolate NB-183 was identified as *V. cholerae* using the *k*-mer-based species identification with Kraken2 database [23] and refseq_masher using Mash MinHash (https://github.com/phac-nml/refseq_masher). The whole-genome sequencing of NB-183 isolate yielded 99 contigs (N_50_ = 125,765 bp) from 4,112,549 bp genome size with genome coverage of 96×, 47.42% G + C content, and BUSCO single-copy completeness of 99.2% (v5.5.0) [13] (Table 1; Datafile 1–2 [24, 25]). Whole genome-based comparison using OrthoANI program [26] showed that NB-183 had average nucleotide identity (ANI) of 98% with *V. cholerae* N16961 (Datafile 1 [24]). In addition, the MLST using the pubMLST database showed that NB-183 had a novel *pyrC* allele and unique allele profile that had been assigned a new sequence type 1668 (Datafile 3, [27]).

**Table 1:** Overview of datafiles/datasets

Label	Name of data file/data set	File types (file extension)	Data repository and identifier (DOI or accession number)
Datafile 1	Table S1, NB-183 – Sequence metrics	MS Excel (.xlsx)	Figshare, 10.6084/m9.figshare.24724467 [24]
Datafile 2	File 1, NB-183 – Genome completeness metrics with BUSCO	Text file (.txt)	Figshare, 10.6084/m9.figshare.24724443 [25]
Datafile 3	Table S2, NB-183 – Multilocus sequence typing	MS Excel (.xlsx)	Figshare, 10.6084/m9.figshare.24844686 [27]
Datafile 4	Table S3, NB-183 – Virulence gene profile	MS Doc (.docx)	Figshare, 10.6084/m9.figshare.25103531 [30]
Datafile 5	Figure S1, NB-183 - Phage regions	Portable Document Format file (.pdf)	Figshare, 10.6084/m9.figshare.24724509 [31]
Datafile 6	Figure S2, NB-183 – Biosynthetic gene cluster regions	Portable Document Format file (.pdf)	Figshare, 10.6084/m9.figshare.24724506 [33]
Dataset 1	Illumina reads of the NB-183 strain	SRA file (.sra)	NCBI Sequence Read Archive, https://identifiers.org/ncbi/insdc.sra:SRR26980564; [22]
Dataset 2	Annotated assembly of the NB-183 strain	Fasta file (.fasta)	NCBI Nucleotide, https://identifiers.org/nucleotide:JAXIPZ000000000 [28]

*V. cholerae* NB-183 strain contained 3,645 protein-coding sequences (CDS), 66 pseudo genes, and 60 RNAs (Dataset 2, [28]). Among the CDS were antibiotic resistance genes *blaCARB*-7 and *almEFG* operon that encode resistance to penams and polymyxin [16, 29], respectively. In addition, 127 virulence genes were identified in the whole-genome, including genes encoding type II secretion system (T2SS) essential components (*espCDEFGHIJKLMN*), type VI secretion system (T6SS)-associated genes (*hcp*-1, *hcp*-2), toxins (*rtxBCD, toxA*), among others. Of note, cholera toxin structural genes (*ctxA* and *ctxB*) and toxin co-regulated pilus gene (*tcpA*) were absent in *V. cholerae* NB-183 (Datafile 4, [30]). No plasmid was detected, but two intact phages (NBp1 and phage NBp2) were identified in the NB-183 genome (Datafile 5, [31]). One of these phages (phage NBp2, size = 6.8 Kbp) was highly similar (97% coverage and nucleotide identity) to *Vibrio* phage VCY-NC_016162.1 [32]. Likewise, six different biosynthetic gene clusters were identified, with only two showing high homology (Blastp = 100%) to two BGCs (vibriobactin and piscibactin) that were identified previously in *Vibrio* species (Datafile 6, [33]). The remaining four BGCs had low homology (Blastp = 0–33%) to those in the Minimum Information about a Biosynthetic Genes Cluster (MIBiG database).

## Limitations

This data note was limited to the description of draft genome of a *V. cholerae* strain isolated from a freshwater sample. Further analysis on a larger collection is needed to source attribute the strain and assess the widespread and significance of the unique biosynthetic gene clusters identified.

## Data Availability

The genomic sequence data described in this Data note has been deposited and freely accessible at DDBJ/ ENA/GenBank. The raw reads were deposited under SRA accession number SRR26980564. The genome annotation version described here is version JAXIPZ000000000.1. Associated datafiles are available on Figshare: Sequence quality metrics and average nucleotide identity [24, 25]; multilocus sequence typing and virulome profile [27, 30]; and phage and biosynthetic gene cluster regions [31, 33].

## Abbreviations

TSA: Tryptic Soy Agar
ANI: Average Nucleotide Identity
BGC: Biosynthetic Gene Cluster
CDS: Coding Sequences
DDH: DNA-DNA Hybridization
MIBiG: Minimum Information about a Biosynthetic Genes Cluster
MLST: Multilocus Sequence Typing
PGAP: Prokaryotic Genome Annotation Pipeline
SRA: Sequence Reads Archive
ST: Sequence Type
T2SS: Type II Secretion System
T6SS: Type VI Secretion System
